# MAPt: A Rapid Antibiotic Susceptibility Testing for Bacteria in Environmental Samples as a Means for Bioterror Preparedness

**DOI:** 10.3389/fmicb.2020.592194

**Published:** 2020-11-05

**Authors:** Ronit Aloni-Grinstein, Ohad Shifman, David Gur, Moshe Aftalion, Shahar Rotem

**Affiliations:** Department of Biochemistry and Molecular Genetics, Israel Institute for Biological Research, Ness-Ziona, Israel

**Keywords:** rapid antibiotic susceptibility testing, preparedness, MAPt, environmental samples, bioterror

## Abstract

Antibiotic resistance of bio-threat agents holds major concerns especially in light of advances in methods for engineering pathogens with antibiotic resistance. Preparedness means for rapid identification and prompt proper medical treatment are of need to contain the event and prevent morbidity and spreading of the disease by properly treating exposed individuals before symptoms appearance. Herein, we describe a novel, rapid, simple, specific, and sensitive method named Micro-Agar-PCR-test (MAPt), which determines antibiotic susceptibility of bio-terror pathogens, directly from environmental samples, with no need for any prior isolation, quantification, or enrichment steps. As proof of concept, we have used this approach to obtain correct therapeutic antibiotic minimal inhibitory concentration (MIC) values for the Tier-1 select agents, *Bacillus anthracis*, *Yersinia pestis*, and *Francisella tularensis*, spiked in various environmental samples recapitulating potential bioterror scenarios. The method demonstrated efficiency for a broad dynamic range of bacterial concentrations, both for fast-growing as well as slow-growing bacteria and most importantly significantly shortening the time for accurate results from days to a few hours. The MAPt allows us to address bioterror agents-contaminated environmental samples, offering rational targeted prophylactic treatment, before the onset of morbidity in exposed individuals. Hence, MAPt is expected to provide data for decision-making personal for treatment regimens before the onset of symptoms in infected individuals.

## Introduction

COVID-19 pandemic is a wake-up call for bio-threats preparedness. Tier-1 select pathogens are defined as bio-threat microorganisms possessing a high probability of causing a severe consequence event. These agents, which may cause mass casualties, constitute a matter of public health concern either in scenarios of natural outbreaks or their malicious use as weaponized agents which under certain circumstances may transit to an epidemic or even a pandemic. Accordingly, pathogens that exhibit ease of dissemination, low infection doses, and potential to be weaponized, such as *Bacillus anthracis*, *Yersinia pestis*, and *Francisella tularensis* are all designated as Tier-1 agents.

*Bacillus anthracis* is the causative agent of anthrax, a highly contagious and fatal disease that can infect humans by the cutaneous, gastrointestinal, or respiratory routes (Dixon et al., 1999; Chitlaru et al., 2011). Annually, between 2,000 and 20,000 of human anthrax cases are reported in different parts of the world including India, Pakistan, Bangladesh, Zimbabwe, United States, South Africa, Iran, Turkey, and more (Goal, 2015). *Bacillus anthracis* exists in the soil in the form of spores, notoriously resilient to environmental insults and therefore able to survive for decades. Spores, which represent the infectious form of the bacteria, can potentially be maliciously used as bio-threat (Goal, 2015). Indeed, inhalational anthrax is the most severe form of the disease. Without treatment mortality rates are above 90%. Yet, prompt diagnosis and rapid appropriate antibiotic treatment were shown to lower the rates dramatically (Holly et al., 2006). The Amerithrax incidents in October 2001, where 22 cases of anthrax were recorded, of which five ended in death, are an example of the bioterror potential of *B. anthracis*.

*Yersinia pestis* is the causative agent of plague, a severe and rapidly progressing lethal disease. *Yersinia pestis* can be transmitted from person to person by inhalation. Symptoms usually begin 1–8 days post-exposure, depending on the infecting strain, dose of pathogen, route of contact, and subsequent disease form (bubonic, septicemic, or pneumonic). High mortality rates occur if treatment is not initiated within 18–24 h after onset of symptoms (Pollitzer, 1954; Inglesby et al., 2000). As such, similar to *B. anthracis*, *Y. pestis* represents a major concern of bioterror use. A deliberate release of 50 kg of aerosolized *Y. pestis* over a city of 5 million may result in 150,000 infected people of which 36,000 are expected to die (WHO, Group of Consultants, 1970). Notably, inoculated *Y. pestis* was shown to survive in the soil for at least 40 weeks (Ayyadurai et al., 2008) and in water bottles for more than 74 days (Torosian et al., 2009). Moreover, it was reported that *Y. pestis* can survive for at least 24 days in contaminated soil under natural conditions (Eisen et al., 2008), hence creating a potentially natural-born contaminated area in case of the death of a sporadically infected animal. Plague is present in different parts of the world; recent outbreaks were reported in Uganda (Respicio-Kingry et al., 2016), China (Shi et al., 2018), Democratic Republic of Congo (Abedi et al., 2018), and Madagascar (Andrianaivoarimanana et al., 2019).

*Francisella tularensis*, the causative agent of tularemia, is highly infectious and can be spread by aerosols. Inhalation of as few as 10 cfu may cause disease (Saslaw and Eigelsbach, 1961; Saslaw et al., 1961). Tularemia has six clinical manifestations: ulceroglandular, glandular, oculoglandular, oropharyngeal, typhoid, and pneumonic (Anda et al., 2007). Pneumonic tularemia is the most relevant in a bioterror scenario as mortality may attain a 60% rate without proper treatment (Gill and Cunha, 1997). Modeling a deliberate release of aerosolized *F. tularensis* over London estimated 2.4 million exposures, 130,000 infections, and 24,000 deaths (Egan et al., 2011). Natural-born infection may arise from bites of infected arthropod vectors, handling of sick or dead animals, inhalation of particles of contaminated soil/dust or vegetation, or consumption of infected food or water (Dennis et al., 2001; Hennebique et al., 2019). *Francisella tularensis* may survive in the environment (water or mud) for a year (Parker et al., 1951). Infections were reported in individuals following lawn mowing (Feldman et al., 2003) and following accidental exposure to contaminated water bodies (Ughetto et al., 2015), thus environmental contamination with *F. tularensis* poses an important public health concern in endemic areas.

All three diseases described above can be treated by antibiotics; no safe and efficient vaccines are currently available for tularemia (Boisset et al., 2014) or plague (Pechous et al., 2016; Demeure et al., 2019). In the case of anthrax, post-exposure treatment relies both on vaccination and antibiotics administration, essential for lowering the bacterial load before the mounting of an efficient anti-anthrax humoral response. Although antibiotics are the first treatment of choice, one should bear in mind that high levels of fluroquinolone-resistant *F. tularensis* mutants can be easily and quickly obtained (Sutera et al., 2014) and some may even share cross-resistance to other clinically-relevant antibiotic classes. Likewise, *B. anthracis* isolates resistant to the recommended antibiotics for prophylaxis and treatment can emerge *in vitro* (Brook et al., 2001; Athamna et al., 2004). Similarly, plasmid-mediated single and multiple drug-resistant strains of *Y. pestis* have been isolated from patients (Guiyoule et al., 2001; Galimand et al., 2006) and non-virulent isolates with reduced susceptibility to ciprofloxacin or doxycycline were produced *in vitro* (Steinberger-Levy et al., 2016; Shifman et al., 2019). These studies underline the essentiality of antimicrobial susceptibility testing (AST) of the infecting bacteria for designing prompt and adequate treatment, which in the case of bioterror agents is mandatory for the management and logistics of the response of the public health authorities. Different approaches have been applied for the rapid identification of suspected pathogens (Maugeri and Lychko, 2019) as well as pathogens of unknown identity (Israeli et al., 2019). However, one should bear in mind that while for identification purposes there is no need for live bacteria, ASTs require that the tested pathogen preserves its viability, adding complexity to the handling of contaminated samples. The conditions for performing AST for these bacteria are defined by the CLSI guidelines (CLSI, 2015) and are based on the microdilution technique for all three agents. Actually, most of the reported studies describe the identification and AST of suspected bacteria from clinical samples of infected individuals. However, as infectious pathogens usually require an incubation period before symptoms emerge infected individuals may infect unknowingly other individuals within their surroundings. Thus, the ability to identify the bacteria and perform an AST, from environmental samples collected from the site of bacteria dissemination, before the onset of clinical symptoms in exposed individuals, holds a great advantage for preparedness, promising early medical treatment of the suspected infected individuals and restriction of the infection chain.

Since inoculum quantity is an important parameter influencing ASTs performance, a defined concentration of a pure culture is required. Accordingly, isolation, enrichment, and quantification steps are essential prior to the AST itself. These procedures are time-consuming and may take several days, depending on the generation time exhibited by the bacterial strain. Taking into account the time required to isolate the bacteria and perform a standard AST – 24 h for *B. anthracis*, 24–48 h for *Y. pestis*, and 48–96 h for *F. tularensis*, there is a need for the development of a faster AST. Indeed, we (Aloni-Grinstein et al., 2015) and others (Weigel et al., 2010) have previously used a quantitative PCR (qPCR) step to monitor growth as a rapid detection method, yet, no alternative for the time-consuming isolation, enrichment, and quantification steps was offered for complex samples. Herein, we describe the development of a rapid and simple AST procedure, denoted Micro-Agar-PCR-test (MAPt), based on a micro-agar dilution test combined with a sensitive and specific qPCR step that circumvents the need to isolate the target bacteria from the contaminated environmental organisms. The described novel procedure dramatically reduces the overall time of the entire procedure. Moreover, the procedure is independent of bacterial concentration and can be implemented for a broad range of bacterial concentrations as low as 10^4^ cfu/ml and high as 10^8^ cfu/ml. To the best of our knowledge, this is the first AST reported to exhibit these advantages, thus allowing to address bioterror agents-contaminated environmental samples before the onset of morbidity in exposed individuals.

In summary, the present report documents a proof-of-concept study for MAPt, for rapid determination of minimal inhibitory concentration (MIC) of Tier-1 agents: *B. anthracis* (5 h), *Y. pestis* (10 h), and *F. tularensis* (16 h). The MAPt assay was applied to various reconstituted environmental samples, spiked with *B. anthracis*, *Y. pestis*, and *F. tularensis*, yielding adequate MIC values within an unprecedentedly short time frame. MAPt may serve as a means for preparedness in a bioterror attack or a natural emerging infectious outbreak.

## Materials and Methods

### Bacterial Strains, Media, and Growth Conditions

The *F. tularensis* live vaccine strain (LVS, ATCC 29684) was grown on Cystine Heart Agar (5.1% CHA supplemented with 1% hemoglobin, Difco) or in cation-adjusted Mueller-Hinton broth (CAMHB; BBL, 212322), supplemented with 2% defined growth supplement (IsoVitaleX Enrichment; BBL 211876) and 3 μM hematin (Sigma 3281) – termed HLMHI – at 37°C. The *Y. pestis* strain EV76 (Ben-Gurion and Shafferman, 1981) was grown on Brain Heart Infusion agar (BHI-A; BD Difco 241830) plates at 28°C. *Bacillus anthracis* Vollum ∆pXO1 ∆pXO2 (Levy et al., 2014) was grown on BHI-A at 37°C. Colony forming units (cfu) counts were determined by platting 100 μl of serial tenfold dilutions in phosphate-buffered saline (PBS, Biological Industries, Beth Haemek, Israel) on CHA for *F. tularensis* and BHI-A plates for *B. anthracis* and *Y. pestis*.

### Preparation of MAPt Plates

Micro-Agar-PCR-test was performed using MHA (BD 225250) for *B. anthracis* and *Y. pestis* and CHA for *F. tularensis.*

Agar media was prepared according to the manufacturer’s instructions. Agar dilution was performed essentially as described in CLSI standard M07 (CLSI, 2018). Following autoclaving, the agar was chilled to 50°C and 40 ml were aliquoted to 50 ml tubes where the tested antibiotic doxycycline (Sigma D9891) and ciprofloxacin (ciproxin 200, Bayer) was added. 10X of antimicrobial solution was diluted by making twofold serial dilutions in master tubes. Next, one part of the 10X antimicrobial solution was added to nine parts of molted agar. Agar with no antibiotics served as growth control. 150 μl aliquots of the antibiotic-supplemented melted agar were divided into a 96-well plate.

### Environmental Sample Collection

Environmental samples were collected from different areas in Israel, from different surfaces, at different weather conditions, representing vast microbial content. No specific permissions were required for the acquisition of the environmental samples, as all collections were performed on unprotected public land. These environmental studies did not involve any endangered or protected species.

The samples were collected from a 20 cm^2^ area using two PBS-damped swabs and one dry swab. The swabs were vortexed vigorously in 5 ml PBS and then discharged. The 5 ml environmental sample was then spiked with the target bacteria at various concentrations. The microorganisms loads in the environmental samples were evaluated by cfu counts. 100 μl of serial 10-fold dilutions in PBS of the environmental sample prior to spiking with the target bacteria, were plated on BHI-A plates and incubated for 24 h at 37°C. The ratio of the contaminant bacteria to the target bacteria was calculated by dividing the cfu of the contaminants to the cfu of the spiked bacteria.

### MAPt Assay

Ten microliters of the tested environmental sample and a 1:10 dilution of the tested sample (in duplicates) were plated in different wells of the MAPt plates containing different concentrations of the tested antibiotics. The MAPt plates were incubated at the optimal growth temperature for each bacteria (28°C for *Y. pestis* and 37°C for *B. anthracis* and *F. tularensis*) for the time required for each bacteria. Of note, a 28°C incubation temperature for *Y. pestis* better supports bacterial growth without affecting the MIC (Frean et al., 2003; Hernandez et al., 2003; Heine et al., 2015), which led to shorter AST durations and easier MIC determination.

### Extraction of Bacteria From MAPt Plates

At the end of the incubation period, 150 μl of PBS was added to each well to recover the bacteria growing on the agar surface. 100 μl of the recovered bacteria was added to 100 μl of Triton buffer (20% Triton-X-100 in TE, Sigma) and the samples were heated for 30 min at 100°C in order to sterilize the sample and extract the DNA. A sample of 5 μl was analyzed by qPCR using a 7500 Real-Time PCR system (Applied Biosystems).

### qPCR Reaction

The qPCR reactions were performed in 30 μl volume containing 2.3 μl of 20 mg/ml bovine serum albumin (BSA; Sigma A2153), 15.05 μl SensiFAST Probe Lo-ROX Mix (Bioline BIO84005), 3.05 μl forward primer (5 pmol/μl), 3.05 μl reverse primer (5 pmol/μl) and 1.55 μl TaqMan probe (5 pmol/μl), and 5 μl of DNA extract. The primers and probes used were as follows:

For *B. anthracis*, the chromosomal marker targeting prophage lambdaBa03 (PL3; Wielinga et al., 2011),

PL3_F: AAAGCTACAAACTCTGAAATTTGTAAATTG.

PL3_R: CAACGATGATTGGAGATAGAGTATTCTTT.

Tqpro_PL3: FAM-AACAGTACGTTTCACTGGAGCAAAATCAA-BHQ-1.

For *Y. pestis*, the gene *capR* encoding the Lon ATP-dependent serine protease (Steinberger-Levy et al., 2016),

capF: GGATTACGATCTCTCGGATGTGA.

capR: AGCCGGACAGACGAATAACTTC.

Taq-CapR: FAM-TTGTGGCGACCTCTAACTCCATGAATATTCC-BHQ-1.

For *F. tularensis*, the gene *fopA* encoding for an outer membrane protein (Versage et al., 2003),

fopAF: ATCTAGCAGGTCAAGCAACAGGT.

fopAR: GTCAACACTTGCTTGAACATTTCTAGATA.

fopAP: FAM-CAAACTTAAGACCACCACCCACATCCCAA-BHQ-1.

The PCR thermal conditions were as follows: 3 min at 60°C followed by 40 cycles of 15 s at 95°C and 35 s at 60°C.

### Quantification of Bacterial Growth Inhibition by qPCR

Bacterial quantification by qPCR was determined using the Ct value, which was extracted by the 7500 real-time PCR system Sequence Detection Software (version 1.4). The relative difference in bacterial growth between an untreated control and antibiotic-treated sample (designated as FC) was calculated by the formula FC = 2^-ΔCt^ where ΔCt is the difference between the Ct of sampled bacteria compared to the Ct of the untreated control sample. A 10-fold change between the antibiotic-treated and untreated samples is reflected by a ΔCt = 3.3 (log_2_10), in an efficient PCR. The MIC was defined as the lowest antibiotic concentration that reduced growth to ΔCt ≥ 3.3, which correlated with the lack of visible growth by the standard AST.

### MIC Determination by Broth Microdilution

Standard broth microdilution was performed according to the CLSI guidelines (CLSI, 2010), in CAMHB for *B. anthracis* and *Y. pestis* and HLMHI for *F. tularensis*. An inoculum of 5 × 10^5^ –1 × 10^6^ cfu/ml suspended in CAMHB for *B. anthracis* and *Y. pestis* and 2 × 10^6^ cfu/ml for *F. tularensis* was added at a 1:1 volume to a 96-well plate (TPP, Cat# 92696) containing duplicates of two-fold serial dilutions of doxycycline (Sigma D9891) or ciprofloxacin (ciproxin 200, Bayer) in CAMHB or HLMHI at a final volume of 0.1 ml. Bacteria grown in CAMHB or HLMHI without the addition of doxycycline or ciprofloxacin served as growth controls in each assay. The 96-well plate was incubated at 37°C for 20 h for *B. anthracis*, 28°C for 24 h for *Y. pesitis* and 37°C for 48 h for *F. tularenis* in an Infinite 200 plate reader (TECAN), and growth was monitored by measuring the optical density at 630 nm (OD630) at 1-h intervals. A 28°C incubation temperature for *Y. pestis* was used because it better supported bacterial growth without affecting the MIC (Frean et al., 2003; Hernandez et al., 2003; Heine et al., 2015), which led to shorter AST durations and easier MIC determination. The MIC values were defined as the lowest doxycycline and ciprofloxacin concentrations that reduced growth to less than 10% of the OD630 measured for the growth control. No growth was verified by visual inspection.

## Results

Micro-Agar-PCR-test is based on the combination of two assays: (a) an agar-dilution method that uses dilutions of antibiotics in agar media to determine MIC values by visual examination of the growth inhibition of bacterial colonies, and (b) a qPCR step which substitutes the visual examination by quantification of the bacterial DNA content of the bacteria. Since the visual appearance of bacterial colonies is time-consuming (overnight for fast-growing bacteria and a couple of days for slow-growing bacteria), we chose to substitute the unaided eye visualization with the qPCR procedure. Furthermore, qPCR increases sensitivity allowing quantification of relatively low amounts of bacteria (~10^4^ cfu/ml). This permitted us to perform the MAPt with relatively low-concentration bacterial samples, which is not applicable by the standard ASTs and to shorten the time of incubation that is need for an answer. Moreover, the use of agar medium better supports the growth of low bacterial concentrations which could not be achieved by using the broth microdilution-based assays.

### MAPt Is Applicable to a Wide Range of Bacterial Concentrations

**Figure 1:**
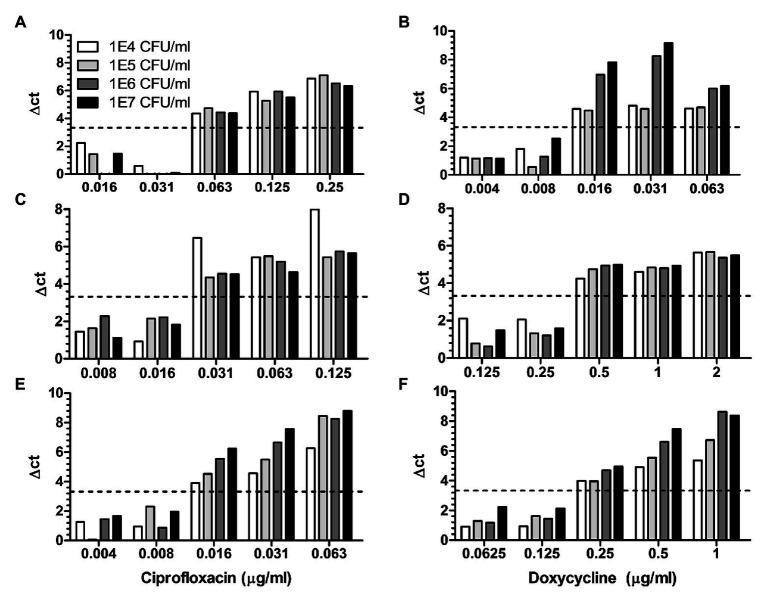
Micro-Agar-PCR-test (MAPt) is applicable to a wide range of bacterial concentrations. Cultures of *Bacillus anthracis* (panels **A**,**B**), *Yersinia pestis* (panels **C**,**D**), and *Francisella tularemia* (panels **E**,**F**), at various concentrations, were subjected to MAPt testing doxycycline and ciprofloxacin. The *B. anthracis* plates were incubated for 5 h and *F. tularensis* plates for 12 h at 37°C and *Y. pestis* plates for 8 h at 28°C. Following incubation the bacteria were harvested from the MAPt plate and subjected to qPCR quantification. Minimal inhibitory concentration (MIC) was determined, as described in materials and methods, by the thresholds of ∆CT = 3.3.

As a preliminary step to setup the MAPt, the minimal time required to detect by qPCR two orders of magnitude of bacterial growth was determined. This 2 log_10_ gap of growth is sufficient to allow the detection of growth inhibition when an efficient antibiotic is tested. We observed that 5 h are required for *B. anthracis*, 8 h for *Y. pestis*, and 12 h for *F. tularensis* for this gap of growth to occur thus leading to the minimal time frame required to detect growth inhibition and to obtain adequate MIC values at a concentration of ~10^6^ cfu/ml (data not shown). Next, we determined if MAPt is applicable to a wide range of initial bacterial concentrations or as in other ASTs is restricted to a defined concentration of bacteria. To that end, 10-fold dilutions of the tested bacteria were subjected to MAPt, and MIC determination was determined as a function of bacterial concentrations. As depicted in Figure 1, MIC values for *B. anthracis* were 0.016 μg/ml for ciprofloxacin and 0.25 μg/ml for doxycycline, for *Y. pestis* 0.031 μg/ml for ciprofloxacin and 0.5 μg/ml for doxycycline, and for *F. tularensis* 0.063 μg/ml for ciprofloxacin and 0.016 μg/ml for doxycycline. These values are in congruence with the MIC values obtained by other ASTs such as E-Tests and microdilution (*B. anthracis*: ciprofloxacin 0.063–0.125 μg/ml, doxycycline 0.016–0.3 μg/ml, *Y. pestis*: ciprofloxacin 0.016–0.064 μg/ml, doxycycline 0.5–1 μg/ml and *F. tularensis:* ciprofloxacin 0.008–0.016 μg/ml and doxycycline 0.125–0.5 μg/ml) and were obtained for all the tested bacterial inoculum concentrations, ranging from 10^4^ to 10^7^ cfu/ml. These results indicate that the MAPt is applicable to a wide range of initial bacterial concentrations when used on pure uncontaminated bacterial cultures.

### MIC Determination of Spiked Environmental Samples

The sensitivity and specificity of MAPt together with the ability to conduct the assay without prior determination of the bacterial load prompted us to challenge the test and evaluate whether MAPt can be applicable also to contaminated environmental samples. It should be noted that the area of a well in the 96-well MAPt plate (32 mm^2^) may provide growth as a monolayer for up to ~4 × 10^7^ cfu of bacteria (based on an assumed area of 0.785 μm^2^ for a single bacteria) suggesting that samples containing up to ~4 × 10^9^ cfu/ml (in a 10 μl drop) can be tested per well. Importantly, the bacterial growth as a monolayer on agar permits the testing of each bacteria as an individual as opposed to the bacterial community tested in the liquid-based microdilution test. Moreover, the specific primers detect only the target bacteria thus providing a specific MIC value even in the presence of contaminating organisms.

### *Bacillus anthracis*-Spiked Environmental Samples

**Figure 2:**
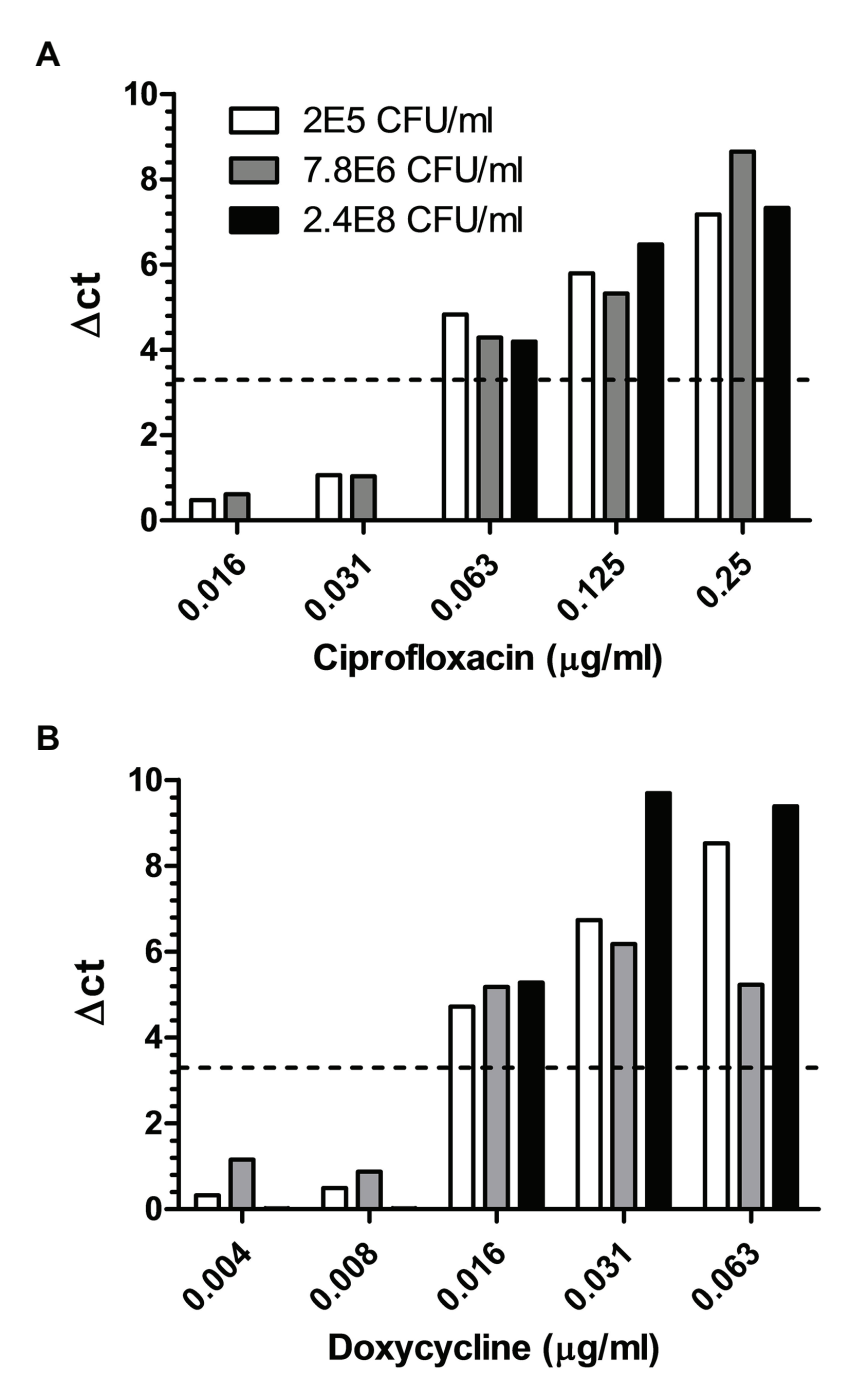
Soiled samples spiked with different concentrations of *B. anthracis* spores. Different soiled samples spiked with 2.4E8, 7.8E6, and 2E5 cfu/ml of *B. anthracis* were subjected to MAPt. MIC was determined, as described in materials and methods, by the thresholds of ∆CT = 3.3. (A) Ciprofloxacin and (B) Doxycycline.

Considering all the above advantages, we examined whether an adequate MIC value can be obtained with environmental samples spiked with *B. anthracis* spores without any prior isolation steps. Diverse environmental samples were collected, outdoors and indoors, from different locations at various weather conditions, thus maximizing the potential of sampling a variety of contaminants. Table 1 describes the various environmental samples, the load of contaminants versus the concentration of the spiked *B. anthracis* spores, and the MIC values obtained by MAPt. Spiked samples varied from as low as 2 × 10^5^ up to 2.4 × 10^8^ cfu/ml *B. anthracis*. Adequate MIC values, for both ciprofloxacin and doxycycline, were obtained for all environmental samples tested. Similar MIC values were obtained, by broth microdilution, for this strain of *B. anthracis*, prior to spiking into the environmental samples (data not shown). Figure 2 represents three independent soil samples (out of the ones listed in Table 1) spiked with different concentrations of *B. anthracis* spores. Each sample was examined in duplicates. Both duplicates yielded proper MIC values of 0.063 μg/ml for ciprofloxacin (Figure 2A) and 0.016 μg/ml for doxycycline (Figure 2B). Soil samples were chosen as representatives since these samples are the most challenging ones as they contain the highest number of contaminating microorganisms. Notably, the soil samples contained high amounts of naturally occurring *Bacillus* spp. phylogenetically close to *B. anthracis* such as *Bacillus cereus*, *Bacillus megaterium*, *Bacillus thuringensis*, and *Bacillus subtilis* (identified by MALDI-TOF, unshown data), at ratios as low as 1:2 to the spiked *B. anthracis* spores, and yet, due to specificity of the assay, owing to the primer used for the PCR which recognizes only *B. anthracis* (Wielinga et al., 2011) adequate MIC values were obtained without the need for any time-consuming isolation/purification steps. All in all, MAPt was shown to be a rapid and reliable AST method for environmental samples containing *B. anthracis* spores within a remarkably short time frame, without the need for any prior isolation steps.

**Table 1:** MIC values obtained by MAPt for *B. anthracis*-spiked environmental samples.

Sample	Concentration of bacteria in the sample (cfu/ml)		Ratio of contaminants to *B. anthracis*	MIC by MAPt (μg/ml)	
	*B. anthracis*	contaminants		Cipro.	Doxy.
**Outdoor samples**					
Soil	2 × 10^5^	5 × 10^4^	1:4	0.063	0.016
Soil	2 × 10^5^	1.1 × 10^5^	1:1.8	0.063	0.016
Soil	6 × 10^5^	7.6 × 10^4^	1:7.9	0.031	0.016
Soil	7.8 × 10^6^	6.6×10^4^	1:78	0.063	0.031
Soil	7.8 × 10^6^	10^5^	1:110	0.063	0.016
Soil	2 × 10^7^	1.1 × 10^5^	1:180	0.063	0.016
Soil	2 × 10^7^	5 × 10^4^	1:410	0.063	0.016
Soil	2.4 × 10^8^	10^5^	1:240	0.063	0.016
Soil	2.4 × 10^8^	6.6 × 10^4^	1:3.6 × 10^3^	0.063	0.016
Asphalt	2 × 10^5^	2 × 10^3^	1:10^2^	0.063	0.016
Asphalt	10^6^	5.33 × 10^2^	1:1.8 × 10^3^	0.063	0.016
Asphalt	2 × 10^7^	2 × 10^3^	1:10^4^	0.063	0.016
Grass	2 × 10^5^	2.4 × 10^4^	1:8.3	0.063	0.016
Grass	10^6^	7.6 × 10^4^	1:13	0.063	0.016
Grass	2 × 10^7^	2.4 × 10^4^	1:830	0.063	0.016
Outdoor stairs	6 × 10^5^	1.1 × 10^4^	1:54	0.031	0.016
**Indoor samples**					
Floor	10^6^	4.6 × 10^3^	1:2.1 × 10^2^	0.063	0.016
Floor	3.5 × 10^6^	7.4 × 10^2^	1:4.7 × 10^3^	0.063	0.016
Door knob	10^6^	5 × 10^3^	1:2 × 10^2^	0.063	0.016
Wall	3.5 × 10^6^	10	1:3.5 × 10^5^	0.063	0.016
Computer keyboard	3.5 × 10^6^	3.6 × 10^2^	1:9.7 × 10^3^	0.063	0.016
Air condition airway	3.5 × 10^6^	10^2^	1:3.5 × 10^4^	0.063	0.016

Asphalt road and soil samples were taken from different locations. MAPt plates were incubated for 5 h at 37°C. Each sample was evaluated in duplicates.

### *Yersinia pestis*-Spiked Environmental Samples

**Figure 3:**
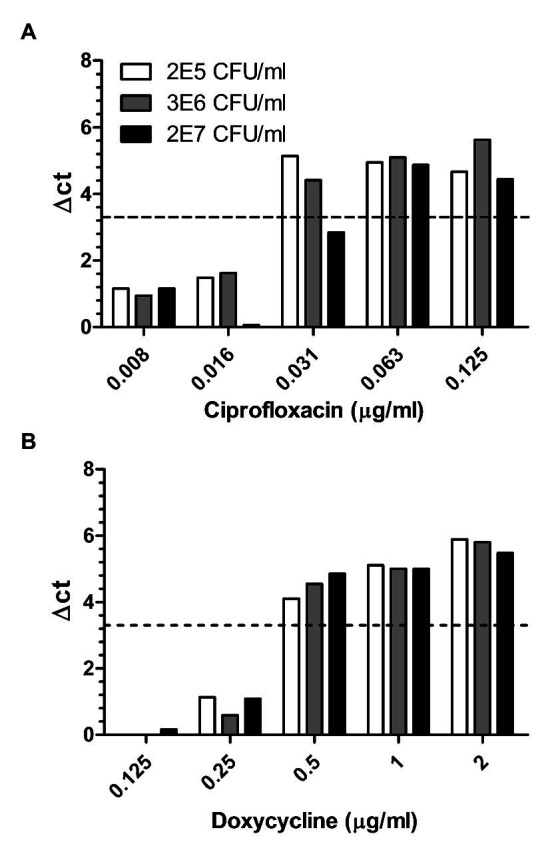
Environmental samples spiked with different concentrations of *Y. pestis*. Different environmental samples spiked with 2E7, 3E6, and 2E5 cfu/ml of *Y. pestis* were subjected to MAPt. MIC was determined, as described in materials and methods, by the thresholds of ∆CT = 3.3. (A) Ciprofloxacin and (B) Doxycycline.

Despite its ability to rapidly cause disease upon infection, *in vitro*, *Y. pestis* is a slow-growing bacteria. Thus, antibiotic susceptibility testing of clinical samples, which depends on the significant growth of the bacteria in culture is often much slower than disease progression. Therefore, the ability to determine antibiotic susceptibility before morbidity bears clinical value. As for *B. anthracis* diverse outdoors as well as indoor environmental samples were collected and subjected to MAPt testing. Table 2 describes the various environmental samples, the load of contaminants versus the concentration of the spiked *Y. pestis*, and the MIC values obtained by MAPt for each sample. Spiked samples varied from as low as 2 × 10^5^ up to 2 × 10^7^ cfu/ml *Y. pestis*. The ratio of contaminants to *Y. pestis* was as low as 1:3. MAPt MIC values of 0.5–1 μg/ml for doxycycline and 0.016–0.064 μg/ml for ciprofloxacin were obtained, similar to the ones obtained, by broth microdilution, for the tested bacteria before spiking into the environmental sample (data not shown). Notably, soil samples at the ratio of 1:3 contaminants to *Y. pestis*, contained *Bacillus* and other spp. known to exhibit higher growth rates than *Y. pestis*. Nevertheless, these contaminants did not interfere with the MAPt assay due to the relatively short incubation time needed for the MAPt assay (10 h). Figure 3 describes MAPt results of three representative spiked environmental samples (out of the ones listed in Table 2) where each sample was tested in duplicates. The same MIC values were obtained for the different duplicates.

**Table 2:** MIC values obtained by MAPt for *Y. pestis*-spiked environmental samples.

Sample	Concentration of bacteria in the sample (cfu/ml)		Ratio of contaminants to *Y. pestis*	MIC by MAPt (μg/ml)	
	*Y. pestis*	contaminants		Cipro.	Doxy.
**Outdoor**					
Soil	3 × 10^4^	3.5 × 10^4^	1:0.86	0.016	0.5
Soil	3 × 10^4^	2.1 × 10^4^	1:1.42	0.031	0.5
Soil	2 × 10^5^	3 × 10^4^	1:6.6	0.031	1
Soil	2 × 10^5^	6 × 10^4^	1:3.3	0.031	0.5
Soil	3 × 10^6^	3.5 × 10^4^	1:86	0.031	0.5
Soil	3 × 10^6^	2.1 × 10^4^	1:1.4 × 10^2^	0.031	1
Soil	2.2 × 10^7^	3 × 10^4^	1:7.3 × 10^2^	0.031	0.5
Soil	2.3 × 10^7^	6 × 10^4^	1:3.8 × 10^2^	0.063	0.5
Asphalt	3 × 10^4^	2.5 × 10^4^	1:1.2	0.016	0.25
Asphalt	2 × 10^5^	1.5 × 10^3^	1:1.3 × 10^2^	0.031	1
Asphalt	3 × 10^6^	2.5 × 10^4^	1:1.2 × 10^2^	0.031	1
Asphalt	2 × 10^7^	1.5 × 10^3^	1:1.3 × 10^4^	0.031	1
**Indoor**					
Floor	2 × 10^5^	3 × 10^2^	1:6.6 × 10^2^	0.063	1
Floor	2 × 10^7^	3 × 10^2^	1:6.6 × 10^4^	0.031	1
Computer keyboard	3 × 10^4^	55	1:5.4 × 10^2^	0.031	0.5
Computer keyboard	3 × 10^6^	55	1:5.4 × 10^4^	0.031	0.5

Asphalt road and soil samples were taken from different locations. MAPt plates were incubated for 10 h at 28°C. Each sample was evaluated in duplicates.

### *Francisella tularensis*-Spiked Environmental Samples

**Figure 4:**
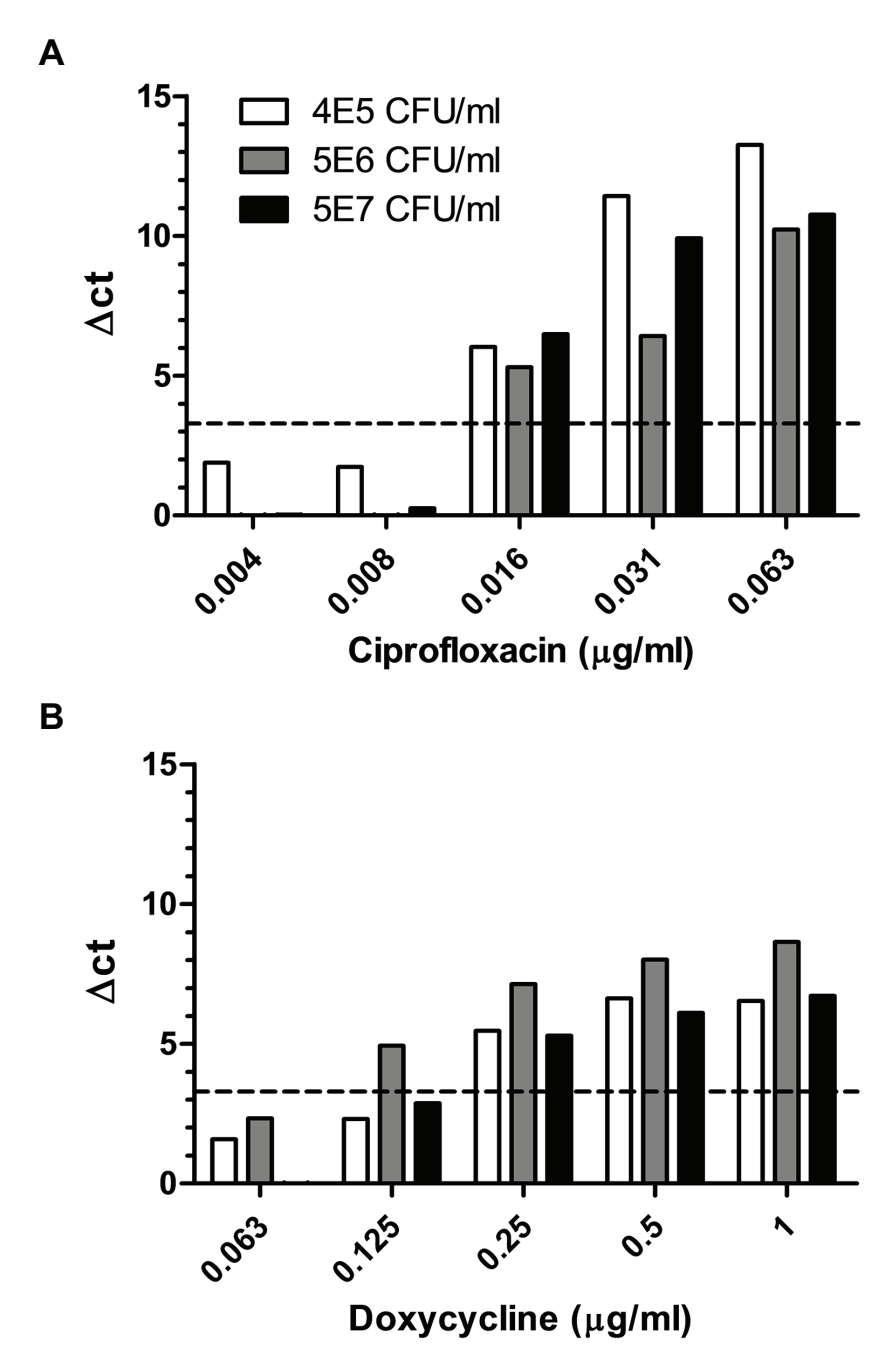
Environmental samples spiked with different concentrations of *F. tularensis*. Different environmental samples spiked with 5E7, 5E6, and 6E5 cfu/ml of *F. tularensis* were subjected to MAPt. MIC was determined, as described in materials and methods, by the thresholds of ∆CT = 3.3. (A) Ciprofloxacin and (B) Doxycycline.

Of the three Tier-1 select agents addressed in this study, *F. tularensis* is the most challenging due to its slow growth rates relative to the contaminants present in the sample. Efforts have been made to improve the efficiency of *F. tularensis* isolation from environmental samples either by the use of defined antibiotics (Petersen et al., 2009) or by acid treatment (Humrighouse et al., 2011). These procedures require a time-consuming culturing step of 2–3 days on the agar plate. Thus, an AST that can generate correct results despite the presence of contaminants is advantageous. Therefore, we tested whether MAPt is adequate to determine the MIC value of *F. tularensis* (samples ranged from 6 × 10^4^ to 5 × 10^7^ cfu/ml of spiked *F. tularensis*) directly from environmental samples. Indeed, as can be seen in Table 3 and Figure 4, owing to the relatively short incubation time and primer specificity characterizing the MAPt assay, the analysis allowed correct determination of proper MIC values of 0.008–0.016 μg/ml for ciprofloxacin and 0.125–0.5 μg/ml for doxycycline for both duplicates of the same sample, similar to those observed by broth microdilution for the tested bacteria before spiking into the environmental sample (data not shown). Taken together, the results demonstrate that MAPt is a rapid AST method for fast as well as slow-growing bacteria.

**Table 3:** MIC values obtained by MAPt for *F. tularensis* spiked environmental samples.

Sample	Concentration of bacteria in the sample (cfu/ml)		Ratio of contaminants to *F. tularensis*	MIC by MAPt (μg/ml)	
	*F. tularensis*	contaminants		Cipro.	Doxy.
**Outdoor**					
Asphalt	6 × 10^4^	13	1:4.6 × 10^3^	0.016	0.125
Asphalt	4 × 10^5^	1.3 × 10^2^	1:4.6 × 10^3^	0.016	0.25
Asphalt	5 × 10^6^	2	1:2.5 × 10^6^	0.016	0.125
Asphalt	5 × 10^7^	20	1:2.5 × 10^6^	0.016	0.25
Soil	6 × 10^4^	4 × 10^2^	1:1.5 × 10^2^	0.031	0.5
Soil	5 × 10^6^	5 × 10^2^	1:10^3^	0.016	0.5
Soil	5 × 10^7^	5 × 10^3^	1:10^4^	0.031	0.5
Side walk	6 × 10^4^	2.5 × 10^2^	1:2.4 × 10^2^	0.016	0.125
Side walk	6 × 10^5^	2.5 × 10^3^	1:2.4 × 10^2^	0.008	0.125
Play ground	4.2 × 10^5^	9	1:4.6 × 10^4^	0.016	0.125
Play ground	4.2 × 10^6^	90	1:4.6 × 10^4^	0.016	0.125
Outdoor stairs	6 × 10^4^	3.7 × 10^3^	1:16	0.008	0.25
**Indoor**					
Building door knob	4 × 10^6^	10^3^	1:1.4 × 10^3^	0.016	0.125
Restaurant door knob	1.8 × 10^7^	10^2^	1:1.8 × 10^5^	0.016	0.125–0.25
Keyboard	4.6 × 10^6^	2 × 10^3^	1:2.3 × 10^3^	0.016	0.125
Air condition airway	2.8 × 10^6^	1.6 × 10^3^	1:1.8 × 10^3^	0.016	0.25

Asphalt roads and soil samples were taken from different locations. MAPt plates were incubated for 16 h at 37°C. Each sample was evaluated in duplicates.

## Discussion

Bioterror or an emerging infectious disease may lead to catastrophic outcomes including morbidity and mortality as well as to economic, social, and psychological repercussions. Thus, preparedness is essential to prevent and control the spread of disease as well as to properly and promptly treat potentially exposed individuals or patients. Rates of morbidity, mortality, and social anxiety can be dramatically reduced if prophylaxis and treatment are initiated as soon as possible. Indeed, over 10,000 doses of post-exposure prophylaxis, at the cost of 2.5 billion dollars, were distributed after the 2001 Amerithrax event, although only 2–3 ounces of *B. anthracis* spores were used (Rebmann, 2014).

The first step when combating a bacterial pathogen is to determine its identity and then its antibiotic susceptibility. This information allows for targeted and proper treatment. Many approaches have been adopted for pathogen identification, among them biochemical testing, culturing in chromogenic media, MALDI-TOF MS, and molecular diagnostic methods, including hybridization-based detection, immunodiagnostic methods, amplification methods, DNA microarrays, and whole genome sequencing (Varadi et al., 2017). Recently, a rapid method for the identification of an unknown pathogen using a high-throughput sequencing-based approach was described and validated for *B. anthracis* and *Y. pestis* – spiked environmental samples (Israeli et al., 2019). Likewise, many methods were described for AST profiling (Li et al., 2017; Maugeri and Lychko, 2019), yet, they require bacterial identification followed by purification and quantification of the bacteria in the sample. Thus, an isolation step, that will discriminate the target bacteria from environmental microbiological contaminants, and an enrichment step are required. These two prerequisites are time-consuming and may take hours to days depending on the bacteria tested. The development of an AST that eliminates these requirements may hold great advantages. We developed a novel approach, termed MAPt, which combines agar dilution with qPCR detection, yielding a rapid, sensitive, specific, and simple AST. The use of the agar medium which better supports the growth of bacteria at low concentrations together with the use of qPCR provides sensitivity and allows quantification of relatively low amounts of bacteria (~10^4^ cfu/ml). Moreover, the use of specific primers allows MIC determination within a shorter time compared to other means even in the presence of contaminating bacteria.

We show that the MAPt is applicable to different bacteria, Gram-negative as well as Gram-positive, fast-growing as well as slow-growing, from a pure culture or directly from contaminated environmental samples. Indeed, we have challenged the assay with environmental samples spiked with *B. anthracis*, *Y. pestis*, and *F. tularemia*, all Tier-1 bioterror select agents. The environmental samples included outdoor as well as indoor sites, from different locations in Israel, at different weather conditions, representing a broad range of contaminating microorganisms at different ratios to the spiked bacteria. Moreover, we have spiked the samples with a range of four orders of magnitude of the target bacteria establishing that MAPt can be implemented with a wide concentration range, starting from as low as ~10^4^ cfu/ml. This allows the omission of the purification, enrichment, and quantification step required in other AST procedures. Furthermore, we show that even when the ratio of the target bacteria to the contaminated bacteria is 1:1, an adequate MIC value is obtained, implying that the isolation and purification steps, of the target bacteria, are not necessary. Due to the use of PCR the assay is objective and reliable compared to the unaided eye examination used in other methods. MAPt can be designed to test a target bacteria to various antibiotics at the same time within the same assay. Likewise, different bacterial samples may be assayed to the same antibiotic. The assay may also be used manually or could be automated to process multiple samples and may serve as a diagnostic tool in clinical settings as well as in research and development.

Moreover, MAPt can beneficially be used to monitor antimicrobial-resistance microbes in the environment. Efforts are being made to understand the connection between human and animal waste, antimicrobials, and resistant microbes in the environment and its impact on human health. Specialists have pointed to the need to assess the location and amounts of resistant microbes present in environmental samples of water, manure, and soils and the impact of mitigation (Singer et al., 2016). Interestingly, a recent paper suggests that the novel Coronavirus COVID-19 outbreak may have global implications for antimicrobial resistance due to the extensive use of antibiotics and biocides (Murray, 2020). This assumption is based on studies showing that resistant bacteria may rise at extremely low antibiotic concentrations, like those detected in wastewater treatment plants and other environmental sites (Thiele-Bruhn, 2003) and that some resistance mechanisms are shared both by biocides and antibiotics (Webber et al., 2015). Sampling strategies and testing methods to define antimicrobial-resistant microbes in the environment should be evaluated (Executive-Summary, 2018). The current methods rely on culture-based methods such as microdilution and disk-diffusion or molecular methods aiming for the detection of resistant genes. Molecular methods are faster and can be applied to bacteria that are hard to culture, however, the detection of a resistant gene not always correlates with resistance (Luby et al., 2016). On the other hand, culture-based approaches, such as microdilution, permit MIC determination, which allows the detection of stepwise escalations in antibiotic resistance in a specific location (Executive-Summary, 2018). MAPt may bridge together the benefits of both approaches.

In conclusion, we have developed a rapid and simple AST for bacteria in environmental sampling, which circumvents the prerequisite for sample culturing, and purification associated with classical approaches. The method may be applied as a preparedness tool in case of a bioterror event, natural emerging infectious disease, or for surveillance strategies.

## Data Availability Statement

The raw data supporting the conclusions of this article will be made available by the authors, without undue reservation.

## Author Contributions

RA-G, OS, and SR conceived the study and wrote the manuscript. All authors contributed to the article and approved the submitted version.

### Conflict of Interest

The authors declare that the research was conducted in the absence of any commercial or financial relationship that could be construed as a potential conflict of interest. Patent application (IL270342) for the described antibiotic susceptibility test (MAPt) was filed by the Israel Institute for Biological Research.

## References

[ref1] Abedi A. A. Shako J. C. Gaudart J. Sudre B. IIunga B. K. Shamamba S. K. B.. (2018). Ecologic features of plague outbreak areas, democratic republic of the Congo, 2004-2014. Emerg. Infect. Dis. 24, 210–220. 10.3201/eid2402.160122, PMID: 29350136 PMC5782875

[ref2] Aloni-Grinstein R. Shifman O. Lazar S. Steinberger-Levy I. Maoz S. Ber R. (2015). A rapid real-time quantitative PCR assay to determine the minimal inhibitory extracellular concentration of antibiotics against an intracellular *Francisella tularensis* live vaccine strain. Front. Microbiol. 6:1213. 10.3389/fmicb.2015.01213, PMID: 26579112 PMC4630301

[ref3] Anda P. Pearson A. D. Tarnvik A. (2007). “WHO guidelines on Tularaemia” in Epidemic and pandemic alert and response. ed. Tarnvik A. (Geneva: World Health Organization), 21–26.

[ref4] Andrianaivoarimanana V. Piloa P. Wagner D. M. Rakootomanana F. Maheriniaina V. Andrianalimanana S.. (2019). Trends in human plague, Madagascar, 1998-2016. Emerg. Infect. Dis. 25, 220–228. 10.3201/eid2502.171974, PMID: 30666930 PMC6346457

[ref5] Athamna A. Athamna M. Abu-Rashed N. Medlej B. Bast D. J. Rubinstein E. (2004). Selection of *Bacillus anthracis* isolates resistant to antibiotics. J. Antimicrob. Chemother. 54, 424–428. 10.1093/jac/dkh258, PMID: 15205405

[ref6] Ayyadurai S. Houhamdi L. Lepidi H. Nappez C. Raoult D. Drancourt M. (2008). Long-term persistence of virulent *Yersinia pestis* in soil. Microbiology 154, 2865–2871. 10.1099/mic.0.2007/0161540, PMID: 18757820

[ref7] Ben-Gurion R. Shafferman A. (1981). Essential virulence determinants of different *Yersinia* species are carried on a common plasmid. Plasmid 5, 183–187. 10.1016/0147-619x(81)90019-6, PMID: 7243971

[ref8] Boisset S. Caspar Y. Sutera V. Maurin M. (2014). New therapeutic approaches for treatment of tularemia: a review. Front. Cell. Infect. Microbiol. 4:40. 10.3389/fcimb.2014.00040, PMID: 24734221 PMC3975101

[ref9] Brook I. Elliott T. B. Pryor H. I. 2nd Sautter T. E. Gnade B. T. Thakar J. H.. (2001). In vitro resistance of *Bacillus anthracis* sterne to doxycycline, macrolides and quinolones. Int. J. Antimicrob. Agents 18, 559–562. 10.1016/s0924-8579(01)00464-2, PMID: 11738344

[ref10] Chitlaru T. Altboum Z. Reuveny S. Safferman A. (2011). Progress and novel strategies in vaccine development and treatment of anthrax. Immunol. Rev. 239, 221–236. 10.1111/j.1600-065X.2010.00969.x, PMID: 21198675

[ref11] CLSI (2010). Methods for antimicrobial dilution and disk susceptibility testing of infrequently isolated or fastidious bacteria: approved guidelines. 2nd Edn. CLSI document M45-A2. Wayne, PA: Clinical and Laboratory Standards Institute.

[ref12] CLSI (2015). Methods for antimicrobial dilution and disk susceptibility testing of infrequently isolated or fastidious bacteria. 3rd Edn. CLSI document M45-A2. Wayne, PA: Clinical and Laboratory Standards Institute.

[ref13] CLSI (2018). Methods for dilution antimicrobial susceptibility tests for bacteria that grow aerobically. CLSI document M07-11th Edn. Wayne, PA: Clinical and Laboratory Standards Institute.

[ref14] Demeure C. E. Dussurget O. Fiol G. M. Le Guern A. S. Savin C. Pizarro-Cerda J. (2019). *Yersinia pestis* and plague: an updated view on evolution, virulence determinants, immune subversion, vaccination and diagnostics. Genes Immun. 20, 357–370. 10.1038/s41435-019-0065-0, PMID: 30940874 PMC6760536

[ref15] Dennis D. T. Inglesby T. V. Henderson D. A. Bartlett J. G. Ascher M. S. Eitzen E.. (2001). Tularemia as a biological weapon: medical and public health management. JAMA 285, 2763–2773. 10.1001/jama.285.21.2763, PMID: 11386933

[ref16] Dixon T. C. Meselson M. Guillemin J. Hanna P. (1999). Anthrax. N. Engl. J. Med. 341, 815–826. 10.1056/NEJM199909093411107, PMID: 10477781

[ref17] Egan J. R. Hall I. M. Leach S. (2011). Modeling inhalational tularemia: deliberate release and public health response. Biosecur. Bioterror. 9, 331–343. 10.1089/bsp.2011.0004, PMID: 22044315 PMC3223019

[ref18] Eisen R. Petersen J. M. Higgins C. L. Wong D. Levy C. E. Mead P. S.. (2008). Persistence of *Yersinia pestis* in soil under natural conditions. Emerg. Infect. Dis. 14, 941–943. 10.3201/eid1406.080029, PMID: 18507908 PMC2600287

[ref19] Executive-Summary (2018). “Initatives for addressing antimicrobial resistance in the environment: current situation and challenges.”

[ref20] Feldman K. A. Stiles-Enos D. Julian K. Matyas B. I. Telford S. R. Chu M.. (2003). Tularemia on Martha’s Vineyard: seroprevalence and occupational risk. Emerg. Infect. Dis. 9, 350–354. 10.3201/eid0903.020462, PMID: 12643831 PMC2958548

[ref21] Frean J. Klugman K. P. Arntzen L. Bukofzer S. (2003). Susceptibility of *Yersinia pestis* to novel and conventional antimicrobial agents. J. Antimicrob. Chemother. 52, 294–296. 10.1093/jac/dkg363, PMID: 12865386

[ref22] Galimand M. Carniel E. Courvalin P. (2006). Resistance of *Yersinia pestis* to antimicrobial agents. Antimicrob. Agents Chemother. 50, 3233–3236. 10.1128/AAC.00306-06, PMID: 17005799 PMC1610074

[ref23] Gill V. Cunha B. A. (1997). Tularemia pneumonia. Semin. Respir. Infect. 12, 61–67. PMID: 9097380

[ref24] Goal A. K. (2015). Anthrax: a disease of biowarfare and public health importance. World J. Clin. Cases 16, 20–23. 10.12998/wjcc.v3.i1.20, PMID: 25610847 PMC4295216

[ref25] Guiyoule A. Gerbaud G. Buchrieser C. Galimand M. Rahalison L. Chanteau S.. (2001). Transferable plasmid-mediated resistance to streptomycin in a clinical isolate of *Yersinia pestis*. Emerg. Infect. Dis. 7, 43–48. 10.3201/eid0701.010106, PMID: 11266293 PMC2631670

[ref26] Heine H. S. Hershfield J. Marchand C. Miller L. Halasohoris S. Purcell B. K.. (2015). In vitro antibiotic susceptibilities of *Yersinia pestis* determined by broth microdilution following CLSI methods. Antimicrob. Agents Chemother. 59, 1919–1921. 10.1128/AAC.04548-14, PMID: 25583720 PMC4356840

[ref27] Hennebique A. Biosset S. Maurin M. (2019). Tularemia as a waterborne disease: a review. Emerg. Microbes Infect. 8, 1027–1042. 10.1080/22221751.2019.1638734, PMID: 31287787 PMC6691783

[ref28] Hernandez E. Girardet M. Ramisse F. Vidal D. Cavallo J. D. (2003). Antibiotic susceptibilities of 94 isolates of *Yersinia pestis* to 24 antimicrobial agents. J. Antimicrob. Chemother. 52, 1029–1031. 10.1093/jac/dkg484, PMID: 14613959

[ref29] Holly J. E. Bravata D. M. Liu H. Olshen R. A. McDonald K. M. Owens D. K. (2006). Systemic review: a century of inhalational anthrax cases from 1900-2005. Ann. Intern. Med. 21, 270–280. 10.7326/0003-4819-144-4-200602210-00009, PMID: 16490913

[ref30] Humrighouse B. W. Adcock N. J. Rice E. W. (2011). Use of acid treatment and a selective medium to enhance recovery of *Francisella tularensis* from water. Appl. Environ. Microbiol. 77, 6729–6732. 10.1128/AEM.05226-11, PMID: 21803910 PMC3187174

[ref31] Inglesby T. V. Dennis D. T. Henderson D. A. Bartlett J. G. Ascher M. S. Eitzen E.. (2000). Plague as a biological weapon. Medical and public health management. JAMA 283, 2281–2290. 10.1001/jama.283.17.2281, PMID: 10807389

[ref32] Israeli O. Cohen-Gilon I. Zvi A. Lazar S. Shifman O. Levy H.. (2019). Rapid identification of unknown pathogens in environmental samples using a high-througput sequencing-based approach. Heliyon 5:e01793. 10.1016/j.heliyon.2019.e01793, PMID: 31193701 PMC6538980

[ref33] Levy H. Gilnert I. Weiss S. Bar-David E. Sittner A. Schlomovitz J.. (2014). The central nervous system as target of *Bacillus anthracis* toxin independent virulence in rabbits and Guinea pigs. PLoS One 9:e112319. 10.1371/journal.pone.0112319, PMID: 25375158 PMC4223028

[ref34] Li Y. Yang X. Zhao W. (2017). Emerging microtechnologies and automated systems for rapid bacterial identification and antibiotic susceptibility testing. SLAS Technol. 22, 585–608. 10.1177/2472630317727519, PMID: 28850804 PMC5835395

[ref35] Luby E. Ibekwe A. M. Zilles J. Pruden A. (2016). Molecular methods for assessment of antibiotic resistance in agricultural ecosystems: prospects and challenges. J. Environ. Qual. 45, 441–453. 10.2134/jeq2015.07.0367, PMID: 27065390

[ref36] Maugeri G. Lychko I. (2019). Identification and antibiotic-susceptibility profiling of infectious bacterial agents: a review of current and future trends. Biotechnol. J. 14:e1700750. 10.1002/biot.201700750, PMID: 30024110 PMC6330097

[ref37] Murray A. K. (2020). The novel Coronovirus COVID-19 outbreak: global implications for antimicrobial resistance. Front. Microbiol. 11:1020. 10.3389/fmcb.2020.01020, PMID: 32574253 PMC7237633

[ref38] Parker R. R. Steinhaus E. A. Kohls G. M. Jellison W. L. (1951). Contamination of natural waters and mud with *Psteurella tularensis* and tularemia in beavers and muskrates in the northwestern United States. Bull. Natl. Inst. Health 193, 1–161. PMID: 14869929

[ref39] Pechous R. D. Sivaraman V. Stasuli N. M. Goldman W. E. (2016). Pneumonic plague: the darker side of *Yersinia pestis*. Trends Microbiol. 24, 190–197. 10.1016/j.tim.2015.11.008, PMID: 26698952

[ref40] Petersen J. M. Carlson J. Yockey B. Pillai S. Kuske C. Garbalena G.. (2009). Direct isolation of *Francisella* spp. from environmental samples. Lett. Appl. Microbiol. 48, 663–667. 10.1111/j.1472-765X.2009.02589.x, PMID: 19413814

[ref41] Pollitzer R. (1954). Plague.WHO monograph series 22. WHO, Geneva, Switzerland.

[ref42] Rebmann T. (2014). Infectious disease disasters: bioterrorism, emerging infections and pandemics. Available at: https://asprtracie.hhs.gov/technical-resource/223/infectious-disease-disasters-bioterrorism-emng-infections-and-pademics (Accessed October 11, 2020).

[ref43] Respicio-Kingry L. B. Yockey B. M. Acayo S. Kaggwa J. Apangu T. Kugeler K. J.. (2016). Two distinct *Yersinia pestis* populations causing plague among humans in the West Nile region of Uganda. PLoS Negl. Trop. Dis. 11:e0004360. 10.1371/journal/pntd.0004360, PMID: 26866815 PMC4750964

[ref44] Saslaw S. Eigelsbach H. T. (1961). Tularemia vaccine study I. Intracutaneous challenge. Arch. Intern. Med. 107, 689–701. 10.1001/archinte.1961.03620050055006, PMID: 13746668

[ref45] Saslaw S. Eigelsbach H. T. Prior J. A. Wilson H. E. Carhart S. (1961). Tularemia vaccine study II. Respiratory challenge. Arch. Intern. Med. 107, 702–714. 10.1001/archinte.1961.03620050068007, PMID: 13746667

[ref46] Shi L. Yang G. Zhang Z. Xia L. Liang Y. Tan H.. (2018). Reemergence of human plague in Yunnan, China in 2016. PLoS One 13:e0198067. 10.1371/journal.pone.0198067, PMID: 29897940 PMC5999221

[ref47] Shifman O. Steinberger-Levy I. Aloni-Grinstein R. Gur D. Aftalion M. Ron I.. (2019). A rapid antibicrobial susceptibility test for determining *Yersinia pestis* susceptibility to doxycycline by RT-PCR quantification of RNA markers. Front. Microbiol. 10:754. 10.3389/fmicb.2019.00754, PMID: 31040834 PMC6477067

[ref48] Singer A. C. Shaw H. Rhodes V. Hart A. (2016). Review of antimicrobial resistance in the environment and its relevance to environmental regulators. Front. Microbiol. 7:1728. 10.3389/fmicb/2016.01728, PMID: 27847505 PMC5088501

[ref49] Steinberger-Levy I. Shifman O. Zvi A. Ariel N. Beth-Din A. Israeli O., et al. (2016). A rapid molecular test for determining *Yersinia pestis* susceptibility to ciprofloxacin by the quantification of differentially expressed marker genes. Front. Microbiol. 7:763. 10.3389/fmicb.2016.00763, PMID: 27242774 PMC4871873

[ref50] Sutera V. Levert M. Burmeister W. P. Schneider D. Maurin M. (2014). Evolution towards high-level fluoroquinolone resistance in *Francisella* species. J. Antimicrob. Chemother. 69, 101–110. 10.1093/jac/dkt321, PMID: 23963236

[ref51] Thiele-Bruhn S. (2003). Pharmaceutical antibiotic compounds in the soil-a review. J. Plant Nutr. Soil Sci. 166, 145–167. 10.1002/jpln.200390023

[ref52] Torosian S. D. Regan P. M. Taylor M. A. Margolin A. (2009). Detection of *Yersinia pestis* over time in seeded bottled water samples by cultivation on heart infusion agar. Can. J. Microbiol. 55, 1125–1129. 10.1139/w09-061, PMID: 19898556

[ref53] Ughetto E. Hery-Arnaud G. Cariou M. E. Pellouz I. Maurin M. Caillon J.. (2015). An original case of *Francisella tularensis* subsp. holarctica bacteremia after a near-drowning accident. Infect. Dis. Ther. 47, 588–590. 10.3109/23744235.2015.1028099, PMID: 25816922

[ref54] Varadi L. Luo J. L. Hibbs D. E. Perry J. D. Anderson R. J. Oernga S.. (2017). Methods for the detection and identification of pathogenic bacteria: past, present and future. Chem. Soc. Rev. 46, 4818–4832. 10.1039/c6cs00693k, PMID: 28644499

[ref55] Versage J. L. Severin D. D. Chu M. C. Petersen J. M. (2003). Development of a multitarget real-time TaqMan PCR assay for enhanced detection of *Francisella tularensis* in complex specimens. J. Clin. Microbiol. 41, 5492–5499. 10.1128/jcm.41.12.5492-5499.2003, PMID: 14662930 PMC309004

[ref56] Webber M. A. Whitehead R. N. Mount M. Loman N. J. Pallen M. J. Piddock L. J. V. (2015). Parallel evolutionary pathways to antibiotic resistance selected by biocide exposure. J. Antimicrob. Chemother. 70, 2241–2248. 10.1093/jac/dkv109, PMID: 25953808 PMC4500774

[ref57] Weigel L. M. Sue D. Michel P. A. Kitchel B. Pillai S. P. (2010). A rapid antimicrobial susceptibility test for *Bacillus anthracis*. Antimicrob. Agents Chemother. 54, 2793–2800. 10.1128/AAC.00247-10, PMID: 20439614 PMC2897299

[ref58] WHO, Group of Consultants (1970). Health aspects of chemical and biological weapons. World Health Organization, 98–109.

[ref60] Wielinga P. R. Hamidjaja R. A. Agren J. Knutsson R. Segerman B. Fricker M.. (2011). A multiplex real-time PCR for identification and differentiating *B. anthracis* virulent types. Int. J. Food Microbiol. 145, 137–144. 10.1016/j.ijfoodmicro.2010.07.039, PMID: 20826037

